# The Importance of Individual-Site and System-Wide Community Health Needs Assessments

**DOI:** 10.3389/fpubh.2020.00020

**Published:** 2020-02-14

**Authors:** Thomas Bias, Christiaan Abildso, Emily Sarkees

**Affiliations:** Health Research Center, School of Public Health, West Virginia University, Morgantown, WV, United States

**Keywords:** social determinants of health, physical determinants of health, community health needs assessment, non-profit hospitals, public health, affordable care act

## Abstract

In order to fulfill the Patient Protection and Affordable Care Act's Community Health Needs Assessment requirements, hospital systems sometimes vary in detail between individual hospital sites or locations and performing an assessment for the entire system. This article examines needs assessments and their accompanying implementation plans across a large university hospital system and finds support for conducting assessments at the local site-level but evidence that system-wide approaches may also have significant benefits, especially at the implementation phase. It suggests a hybrid approach to the needs assessment process where systems and their individual hospitals work together to maximize health benefits to the communities served.

## Introduction

Since the inception of the 2010 Patient Protection and Affordable Care Act's (ACA's) requirement for non-profit hospitals to conduct Community Health Needs Assessments (CHNAs) every 3 years, most hospitals should have completed at least two cycles of identifying health needs in the community, developing implementation plans, and working to improve the health of their local service areas. Large hospital systems, made up of multiple individual hospital locations, have differed in their approach to conducting these CHNAs. Some have completed one CHNA report and its accompanying implementation plan for all hospitals within the system whereas others have completed CHNA and implementation plans for each individual hospital within a system. The 2010 ACA required non-profit hospitals across the United States to complete Community Health Needs Assessments whereby the hospital gathers community input and examines other data sources to identify the most important public health issues facing their service areas. After identification, hospitals must choose health issues to prioritize and create implementation strategies to address those needs. This requirement is tied directly to the tax-exempt status of hospitals and must be completed every 3 years (1). Activities related to the CHNA implementation plan are reported on the Internal Revenue Service Form 990. Prior literature has indicated the wide variation of quality among CHNAs, (2) including a lack fo consistency in method and content (3).

While community benefit has been notoriously hard to capture by non-profit hospitals, it was estimated that spending in 2012 was over 60 billion dollars in the United States (4). This enormous influx of money, part of which should now be directed toward significant public health need in communities served by these hospitals, underscores the importance of further understanding best practices for CHNA processes and the need for clarity and consistency in CHNA reporting (5). There is wide variation in the amount of this spending across hospitals, and while research has shown that prior to the CHNA act much of this spending was patient-care related (6), there is also some variation in how sites have moved this toward higher community benefit spending, tied directly to the level of CHNA implementation planning (7).

In light of these large sums of public health spending, the CHNA process has potential to be an important mechanism for improving public health at the population level and addressing systemic and environmental factors, including social determinants of health, that have proven to be difficult problems for public health practitioners to solve (8). Over the past decade there has been a tremendous growth in both the number of hospital systems (more than one hospital formally affiliated with each other) and the number of independent hospitals who have moved toward affiliation with a system (9). Hospital systems have the potential to reach large populations with both healthcare services and public health interventions through the CHNA process. Systems generally complete separate reports for each site affiliated with the hospital system, although in some cases system reports combine one or more sites into one report (10). The presence of one overarching report summarizing needs and implementation strategies across all sites within systems also varies from system to system.

Research has pointed to the importance of collaboration across hospital systems (11) and between hospitals and community partners, both through input from the public (12–14) and with local stakeholders such as health departments (15). The literature also emphasizes that increasing the scope of collaboration can help increase the resources brought to bear on projects and the benefits of expanded partnerships (11) and regional coordination (16). Potential areas where hospitals and other public partners could share needs assessment data have also been highlighted (3). Using the West Virginia University Medicine hospital system, we identify the variation in responses to the health needs identified by each local hospital siteindependently and determine which health needs were prioritized by each. Further, we attempt to cross-reference implementation strategies across each and discuss the potential for intra-system overlap and collaboration. The findings here, which will lead to a system-wide plan for the specific hospital system, also hold lessons learned for other hospitals who are a part of a larger healthcare system, but potentially also for hospitals who could coordinate or collaborate with other regional hospitals and community partners to extend resource availability for implementation around common public health goals.

## Materials and Methods

We examined eight hospitals within the West Virginia University Medicine system who went through a nearly-identical process of CHNA within the past 5 years. Table 1 describes each hospital.

**Table 1:** Descriptive statistics of eight hospitals affiliated with the WVU medicine system.

**Hospital (year of last completed CHNA)**	**Number of counties in self-defined service area**	**Total population of counties in service area**	**Number of beds**
Barnesville Hospital (2016)	5	152,597	25
Camden Clark Medical Center (2017)	10	252,318	302
Jefferson/Berkeley Medical Centers (2018)	2	168,383	220
Potomac Valley Hospital (2018)	3	128,070	25
Ruby Memorial Hospital (2018)	1	105,030	684
St. Joseph's Hospital (2019)	1	24,415	51
Summersville Regional Medical Center (2018)	1	25,043	90
United Hospital Center	2	76,892	292

For each hospital we indexed all needs prioritized (prioritized needs were not given in order of importance in the reports) and each implementation strategy chosen by the hospital. We adopted the Healthy People 2020 (17) list of social and physical determinants of health and coded each hospital implementation strategy into one of the following categories as subcategories of each identified need (obesity strategies, substance abuse strategies, etc.): *Social Determinants included:*

- Availability of resources to meet daily needs (e.g., safe housing and local food markets)
- Access to educational, economic, and job opportunities
- Access to health care services
- Quality of education and job training
- Availability of community-based resources in support of community living and opportunities for recreational and leisure-time activities
- Transportation options
- Public safety
- Social support
- Social norms and attitudes (e.g., discrimination, racism, and distrust of government)
- Exposure to crime, violence, and social disorder (e.g., presence of trash and lack of cooperation in a community)
- Socioeconomic conditions (e.g., concentrated poverty and the stressful conditions that accompany it)
- Residential segregation
- Language/Literacy
- Access to mass media and emerging technologies (e.g., cell phones, the Internet, and social media)
- Culture.

*Physical determinants* included:

- Natural environment, such as green space (e.g., trees and grass) or weather (e.g., climate change)
- Built environment, such as buildings, sidewalks, bike lanes, and roads
- Worksites, schools, and recreational settings
- Housing and community design
- Exposure to toxic substances and other physical hazards
- Physical barriers, especially for people with disabilities
- Aesthetic elements (e.g., good lighting, trees, and benches).

## Results

Results are presented as % of hospitals who identified and prioritized each need (Table 2) and the number of strategies addressing each need across the system stratified by physical and social determinants of health (Table 3). Since hospitals chose differing numbers of health needs to prioritize and differing numbers of strategies to address each need, the implementation planning numbers in Table 3 can represent more than one strategy within a hospital.

**Table 2:** Health needs prioritized by hospitals in the WVU medicine system.

**Health need prioritized in plan**	**% of hospitals prioritizing this need**
Obesity and related chronic disease (diabetes, heart disease, etc.)	100
Substance use	87.5
Cancer	62.5
Smoking and related disease (COPD, asthma, etc.)	37.5
Access to care	25.0
Mental health	25.0

**Table 3:** Implementation strategies chosen by hospitals for each prioritized need.

**Determinants of health addressed by topic** **P indicates physical** **S indicated social**	**Number of strategies addressing across all hospitals**
**Drug Addiction**	
Access to healthcare services (S)	11
Social support (S)	5
Social norms and attitudes (S)	5
Exposure to toxic substances and other hazards (P)	1
**Mental health**	
Access to healthcare services (S)	2
Social support (S)	1
**Smoking and related disease**	
Healthcare services (S)	2
Social support (S)	1
**Obesity and related chronic disease**	
Availability of community-based resources in support for recreational and leisure time activities (S)	6
Availability of resources to meet daily needs (S)	4
Social support (S)	4
Social norms and attitudes (S)	2
Built environment (P)	1
Access to healthcare services (S)	1
**Access to healthcare**	
Access to healthcare services (S)	3
Transportation options (S)	1
**Cancer**	
Access to healthcare services (S)	5
Social norms and attitudes (S)	3

## Discussion

Our scan of CHNAs in the West Virginia University Medicine system indicates substantial overlap in the needs chosen by each hospital in the system. Only six total issues were identified and prioritized across all eight hospitals, with each hospital generally choosing 2–4 strategies each. More than half of the hospitals chose obesity and related disease, cancer, and substance abuse issues indicating these are significant issues across large portions of the system. Three issues (smoking and related disease, access to care, and mental health), however, were chosen by only a few hospitals. These results indicate that there are both substantial overlaps within communities but also strengthen the idea that needs assessment should be done at the local level and not just across the system to identify needs that might exist within smaller pockets of a system's service area and might be best served by an individual hospital site rather than leveraging system resources. A combination of local level data collection [especially considering the need to link local population health data and rankings to the needs selected by individual hospitals (18)] and CHNA reports and a system-wide reporting and implementation planning mechanism would be a strong combination for healthcare systems looking to have an impact on large-scale population health. These findings reiterate findings in the literature speaking to the importance of regional planning for community benefit (16).

Turning to implementation planning, strategies to address needs varied considerably from site to site. This may be a result of the large differences found within the population size of service areas, the geographic reach of the service areas, and the size of hospital (illustrated in Table 1 by the number of beds) which may also indicate the level of resources available at the local level. As implementation efforts continue, it is important to conduct evaluation of the impact of these efforts to determine which have a substantial impact on the needs identified within the communities. The overall hospital system is in a key position to communicate successful and unsuccessful efforts across the system and leverage additional resources toward successful interventions in order to have a stronger impact on the public health of hospital service areas. This may be especially true if resource-intensive strategies to address health concerns are evaluated and seen to have a larger impact. Small (and largely rural) sites may not have the ability to replicate successes of large hospitals due to lack of resources (19).

Across the system, the vast majority (96.6%) of implementation strategies addressed social determinants of health. Only two strategies addressed physical determinants of health. We hypothesize this may be a result of individual sites thinking about minimal resources they may have to leverage toward significant health issues. Coordinating a response across the system may increase the ability to address physical determinants of health, especially with the issue of obesity and related chronic diseases. At the same time, there may be other factors that keep hospitals from pursuing strategies related to physical determinants of health (20), so this issue may require more in-depth study.

These findings also point to a need to replicate a study such as the one conducted by Pennel, et al. (2) to revisit quality of CHNAs across individual hospitals. Further study could also scope out the varied ways systems are reporting individual site needs, implementation strategies, and how many are combining these into an overall system report.

The major limitation of this study was the ability to look at needs identified and priorities selected only among one hospital system. Further, the community health needs assessments and implementation plans were not all conducted by the same individuals or reported in the same format so there was some variation across plans that were compared.

The results presented above demonstrate both the need for individual sites to conduct their own community health needs assessments to identify unique local health issues, but also suggest there may bestrength in a system-wide approach to addressing common regional health needs. Moving forward, hospitals should consider a system-wide report that breaks down individual sites and looks at where the system could have the most impact on significant needs across its population served. Systems could leverage their larger regional resources to help shape the CHNA process developed by the ACA into a powerful public health tool.

## Data Availability Statement

The datasets generated for this study are available on request to the corresponding author.

## Author Contributions

TB took the lead authorship on this manuscript including the conceptual idea for the manuscript, initial drafting, and majority of writing content. ES developed the data for the tables, conducted the data collection, and assisted with overall writing. CA assisted with conceptualizing the tables, wrote portions of the discussion section, and further developed the determinants of health methodology.

### Conflict of Interest

All authors are employees of West Virginia University. The authors have been contracted through the West Virginia University Hospital System to conduct Community Health Needs Assessments in the past and present. The authors declare that the research was conducted in the absence of any commercial or financial relationships that could be construed as a potential conflict of interest.
